# Nonlinear digital signal processing in mental health: characterization of major depression using instantaneous entropy measures of heartbeat dynamics

**DOI:** 10.3389/fphys.2015.00074

**Published:** 2015-03-13

**Authors:** Gaetano Valenza, Ronald G. Garcia, Luca Citi, Enzo P. Scilingo, Carlos A Tomaz, Riccardo Barbieri

**Affiliations:** ^1^Department of Anesthesia, Critical Care & Pain Medicine, Massachusetts General Hospital, Harvard Medical SchoolBoston, MA, USA; ^2^Massachusetts Institute of TechnologyCambridge, MA, USA; ^3^Department of Information Engineering, Research Center E. Piaggio, University of PisaPisa, Italy; ^4^Martinos Center for Biomedical Imaging, Department of Radiology, Massachusetts General HospitalCharlestown, MA, USA; ^5^Department of Psychiatry, Masira Research Institute, Medical School, Universidad de SantanderBucaramanga, Colombia; ^6^School of Computer Science and Electronic Engineering, University of EssexColchester, UK; ^7^Laboratory of Neuroscience and Behavior, Universidade de BrasiliaBrasilia, Brazil

**Keywords:** nonlinear, heart rate variability, instantaneous entropy, point process, Wiener-Volterra series, Laguerre expansion

## Abstract

Nonlinear digital signal processing methods that address system complexity have provided useful computational tools for helping in the diagnosis and treatment of a wide range of pathologies. More specifically, nonlinear measures have been successful in characterizing patients with mental disorders such as Major Depression (MD). In this study, we propose the use of instantaneous measures of entropy, namely the inhomogeneous point-process approximate entropy (ipApEn) and the inhomogeneous point-process sample entropy (ipSampEn), to describe a novel characterization of MD patients undergoing affective elicitation. Because these measures are built within a nonlinear point-process model, they allow for the assessment of complexity in cardiovascular dynamics at each moment in time. Heartbeat dynamics were characterized from 48 healthy controls and 48 patients with MD while emotionally elicited through either neutral or arousing audiovisual stimuli. Experimental results coming from the arousing tasks show that ipApEn measures are able to instantaneously track heartbeat complexity as well as discern between healthy subjects and MD patients. Conversely, standard heart rate variability (HRV) analysis performed in both time and frequency domains did not show any statistical significance. We conclude that measures of entropy based on nonlinear point-process models might contribute to devising useful computational tools for care in mental health.

## Introduction

Rapid developments in healthcare technology render digital signal processing crucial in revealing manifold information regarding human physiological functioning and pathological condition. To date, most of the proposed digital processing techniques consider noninvasive biomedical signals such as the electrocardiogram (ECG), respiration activity, and body movement activity, in order to extract physiological parameters [e.g., mean heart rate (HR) and respiratory frequency] and demonstrate that they can provide clinically relevant information (He et al., 2013).

Over the last two decades, *ubiquitous and pervasive computing* has permeated healthcare (Korhonen and Bardram, 2004) in helping to gather health-related information from wearable or embedded sensors, even in settings outside the hospital. Successful examples have proven to be those systems that monitor the activity and function of the Autonomic Nervous System (ANS) in mental healthcare (see e.g., Agelink et al., 2002; Korhonen and Bardram, 2004; Wittchen and Jacobi, 2005; Lemoult et al., 2012; Yang and Tsai, 2013).

Mathematical modeling and digital signal processing techniques play an important role in the study of cardiovascular control physiology and heartbeat dynamics (Acharya et al., 2006). The cardiovascular system is often investigated through analysis of event series obtained by computing the time intervals between two consecutive R-waves as detected from the ECG, i.e., the RR intervals. Because the heartbeat is controlled by the ANS, the RR interval series shows preferred oscillations around the mean value, defined as Heart Rate Variability (HRV) (Acharya et al., 2006). A recently proposed exemplary case having at its core the digital signal processing of heartbeat dynamics is provided by a multiparametric wearable platform for the physiological/behavioral monitoring of mood fluctuation in bipolar patients (Valenza et al., 2014a,b).

The signal processing methodology behind these ANS/ECG-based healthcare systems is based on linear analysis (performed in both time and frequency domains) aimed at identifying a limited number of oscillatory components to characterize heartbeat dynamics. Nevertheless, the complexity of the cardiovascular control system calls for processing tools able to transcend a simplistic identification. Several nonlinear measures of HRV, in fact, such as Lyapunov exponents, 1/f slope, approximate entropy, and detrended fluctuation analysis, have been widely used to uncover nonlinear fluctuations in HR that are not otherwise apparent (Sunagawa et al., 1998; Mathews and MacLeod, 2005; Nierenberg, 2008; Voss et al., 2009; Leistedt et al., 2011; Soleimani et al., 2011; Rakofsky et al., 2013; Cornforth et al., 2014). Consequently, such measures have provided important quantifiers of cardiovascular control dynamics, mediated by the ANS, and have been found to have prognostic value in aging and disease. Although the detailed physiology behind cardiovascular complex dynamics has not been completely clarified, nonlinear HRV dynamics may be partly explained by the various nonlinear neural interactions and integrations occurring at the neuron and receptor levels, which underlie the complex output of the sinoatrial node in response to changing levels of efferent autonomic inputs (Sunagawa et al., 1998). It is also commonly thought that the complexity of healthy dynamics can be interpreted as an essential part of their ability to adapt to a varying environment.

Depression is a global public health problem of very high prevalence (Nierenberg, 2008) characterized by persistent feelings of sadness and loss of interest or pleasure in daily activities for at least a 2 week period, with other symptoms including psychomotor retardation, fatigue, feelings of worthlessness, and recurrent thoughts of death (Soleimani et al., 2011). Clinical features related to autonomic dysfunction such as decreased appetite, gastrointestinal parestesias, imsomnia, and increased sweating are also frequently reported (Rakofsky et al., 2013). All these alterations seem to be reinforced by a mood-congruent cognitive bias in which MD patients give preference to the processing of negative vs. positive emotional content material (Leistedt et al., 2011). The exposure to negative emotional stimuli easily activates other negative thoughts and memories in MD patients, thus contributing to maintaining and enhancing depressive symptomatology (Mathews and MacLeod, 2005). The proposed analysis has been inspired by several works that relate ANS markers to depression (Mathews and MacLeod, 2005; Nierenberg, 2008; Leistedt et al., 2011; Soleimani et al., 2011; Rakofsky et al., 2013). In particular, it has been shown that linear-derived parameters are quite unreliable in effectively discerning healthy subjects and patients with major depressive disorder, as they have a high inter-subject variability. On the contrary, nonlinear measures such as MultiScale Entropy (MSE) allowed for the discrimination of depressive patients and healthy subjects in always showing a significant decrease of the complexity in the pathological group (Leistedt et al., 2011; Yang and Tsai, 2013; Valenza et al., 2014c).

Nonlinear analysis of HRV data might also be successful in the individual assessment of psychiatric disorders. Most of the known mental disorders, in fact, are currently diagnosed by simply relying on the clinician's experience, possibly supported by verbal interviews and scores from specific questionnaires (Wittchen and Jacobi, 2005). Therefore, a more automated mental assessment through non-invasive, easy-to-record, and robust physiological time series such as HRV could open dramatic clinical perspectives in objectively managing the illness, thereby helping patients, facilitating the interaction between patient and physician, as well as alerting professionals in case of critical pathological episodes.

In this study, we attempt to characterize major depression (MD) by using a recently introduced computational method of nonlinear digital signal processing. Accordingly, we hypothesize that instantaneous nonlinear analysis based on entropy measures can provide useful information about the clinical state of patients with MD while tracking the related complex cardiovascular dynamics. To this extent, we propose the application of point-process nonlinear models of heartbeat dynamics to derive instantaneous indices of complexity. In fact, we recently improved the point-process framework, where the RR interval series is seen as a binary stochastic series characterized by inter-event probability functions (Leistedt et al., 2011), by embedding nonlinear autoregressive models with the Laguerre expansion of the Wiener–Volterra autoregressive terms. In doing so, we both achieved a more effective system identification (Valenza et al., 2014d), and derived novel instantaneous indices of complexity, i.e., the inhomogeneous point-process approximate entropy (*ipApEn*) and the inhomogeneous point-process sample entropy (*ipSampEn*) indices (Valenza et al., 2014d). In this work, after describing the experimental procedures and the basic mathematical formulation related to the point-process nonlinear modeling, we test the effectiveness of the instantaneous entropy measures in distinguishing patients with MD from healthy subjects.

## Materials and methods

### Recruitment of eligible subjects

A number of 48 Hispanic outpatients with MD were included in the study. All subjects were screened in local universities by applying the Zung-self-rating depression scale (Zung, 1965). This scale is a 20-item questionnaire that measures the presence and severity of depressive symptomatology in the preceding 2 weeks. A score of 50/100 was considered compatible with a diagnosis of depression according to previous data in our population (Campo-Arias et al., 2006). A Spanish Structured Clinical Interview for DSM-IV Axis 1 disorders, Clinical version, was applied by qualified psychiatrists to confirm the diagnosis of MD. All patients were experiencing their first MD episode and had not received psychotherapeutic or pharmacological treatment. A control group consisting of 48 age- and gender-matched healthy subjects was also included. Exclusion criteria for both MD and healthy subjects were: cardio-, cerebro-, or peripheral vascular diseases, the presence of neoplasm, diabetes mellitus, kidney or liver failure, infectious or systemic inflammatory disease, and current neurological illnesses. All subjects received information about the study procedures and gave written informed consent approved by the local Institutional Review Board. Data coming from this study was recently published to investigate sex differences in cardiac autonomic function and plasma nitrate levels and endothelial function in MD (García et al., 2011, 2012).

### Experimental protocol and data acquisition

The stimulus used in the study consisted of a set of 11 slides accompanied by an audio recording with two different narrative versions: an emotionally neutral recording (N) and an emotionally arousing one (E). These stories had been previously adapted and validated in a sample from the same Hispanic background (Botelho de Oliveira et al., 2012) and were kept as close as possible to the originals (Cahill and McGaugh, 1995). Both sets of slides showed a mother taking her young son to see his father at a nearby hospital where he works. The slides were identical, but the narrative differed in the N and E versions. In the N version of the story, the mother and son witness a minor car accident, which attracts the attention of the child, whereas in the E version, the child himself is critically injured and requires a surgical intervention at the hospital. The story content can be divided into three phases, with the second phase (slides 5–8) containing the emotionally arousing elements.

All recording sessions took place between 8 a.m. and 10 a.m. All tests were performed in a quiet, dimly lit room at a comfortable temperature (20–22°C). Participants abstained from smoking or consuming beverages containing caffeine, xanthines, or alcohol the day before evaluation. Subjects from each group (MD, HC) were randomly assigned to either undergo the N or E stimulus version, resulting in four different experimental groups (MD-N, MD-E, HC-N, HC-E). All subjects were told that the aim of the study was to evaluate how people pay attention to stories. It was explained that the slide presentation would be shown accompanied by a short narration. They were instructed to concentrate on each slide for the duration of its presentation and to watch the slide show as they would watch a movie. Continuous ECG monitoring (lead II) was performed with a Finometer device (Finapress Medical System, The Netherlands). Data was digitized and stored in a PC computer using a signal acquisition system DATAQ 720-WINDAQ PRO (DataQ Instruments, Akron, OH, USA). Subjects were initially asked to rest for 10 min in a reclining position. Then, each subject underwent the stimulus elicitation previously described, followed by a 3-min recovery period, during which participants quietly rested.

### Point-process modeling of cardiovascular dynamics

Given a single heartbeat event R and the events set {*u_j_*}*^J^_j_* = 1 detected from the ECG, *RR_j_* = *u_j_* − *u*_*j* − 1_ > 0 denotes the *j*th R-R interval within the observation interval *t* ϵ (0, *T*].

Assuming history dependence and an inverse Gaussian probability distribution function (IG-pdf) of the waiting time *t* − *u_j_* until the next R, it is possible to write (Valenza et al., 2014d,e):

(1) $$\begin{array}{l} \;f\left(t|H_{t},\zeta \left(t\right)\right)={\left[\frac{\zeta_{0}\left(t\right)}{2\pi {\left(t-\mu_{j}\right)}^{3}}\right]}^{\frac{1}{2}}\times \\ exp\left\{\frac{1}{2}\frac{\zeta_{0}\left(t\right){\left[t-\mu_{j}\;-\mu_{RR}\left(t,H_{t},\zeta \left(t\right)\right)\right]}^{2}}{\mu_{RR}{\left(t,H_{t},\zeta \left(t\right)\right)}^{2}\left(t-\mu_{j}\right)}\right\} \end{array}$$

With *j* = *Ñ* (*t*) the index of the previous event before time *t* and *Ñ*(*t*) the left continuous sample path of the associated counting process, *H_t_* = (μ_*j*_, *RR_j_*, *RR*_*j* − 1_, …, *RR*_*j* − *M* + 1_) the history of the past heartbeat events, ζ(*t*) the vector of the time-varying parameters, μ_*RR*_(*t, H_t_*, ζ(*t*)) the first-moment statistic (mean) of the distribution, and ζ_0_(*t*) >0 the shape parameter of the inverse Gaussian distribution.

As *f*(*t*|*H_t_*, ζ(*t*)) indicates the probability of having an event at time t given that a previous event has occurred at μ_*j*_ and μ_*RR*_(*t*, *H_t_*, ζ(*t*)) can be interpreted as signifying the most probable moment when the next event could occur. In order to compute the ipApEn and ipSampEn indices, which are described in the next paragraph, we apply a formulation of μ_*RR*_(*t, H_t_*, ζ(*t*)) based on a Nonlinear Autoregressive Model with Laguerre expansions (NARL) of the following terms:

(2) $$\begin{array}{l} \;\mu_{RR}\left(t,H_{t},\zeta \left(t\right)\right)=RR_{\tilde{N}\left(t\right)}+g_{0}\left(t\right)+\\ \sum_{i=0}^{p}g_{1}\left(i,t\right)l_{i}\left(t^{-}\right)+\sum_{i=0}^{q}\sum_{j=0}^{q}g_{2}\left(i,j,t\right)l_{i}\left(t^{-}\right)l_{j}\left(t^{-}\right) \end{array}$$

where *l_i_*(*t*^−^) = $\sum_{n=0}^{p}\Phi_{i}\left(n\right)$ (*RR*_*Ñ*(*t*) − *n*_ − *RR*_*Ñ*(*t*) − *n* − 1_) is the output of the *i*th Laguerre filters Φ_*i*_ just before time *t*. Of note, we process the derivative R-R series to improve on the achievement of stationarity within the sliding time window W (in this study *W* = 90 s).

As a major advantage, the Laguerre filtering allows for a parsimonious number of unknown parameters that need be estimated in Equation (2), and the implementation of a nonlinear autoregressive Volterra–Wiener model with second order nonlinear terms and long-term memory.

Since μ_*RR*_(*t*, *H_t_*, ζ(*t*)) is defined in continuous time, it is possible to obtain an instantaneous R-R mean estimated at an arbitrarily fine timescale, which requires no interpolation between the arrival times of two beats.

Given a time-varying local observation interval of duration W, we find the unknown time-varying parameter vector that maximizes the local log-likelihood through the well-known Newton–Raphson procedure (Valenza et al., 2014d).

The recursive, causal nature of the estimation allows for predicting each new observation given the previous history independently at each iteration. The model and its parameters are therefore also updated at each iteration without priors. We determine the optimal model order {p, q} based on the Akaike Information Criterion and the model goodness-of-fit (obtained by prefitting the model to a subset of the data), which is based on the Kolmogorov–Smirnov (KS) test and associated KS statistic (Valenza et al., 2014e). Autocorrelation plots are also considered to test the independence of the model-transformed intervals (Valenza et al., 2014e). Once the order {p, q} is determined, the initial NARL coefficients are estimated by the method of least squares (Valenza et al., 2014e).

### Instantaneous entropy estimation: the inhomogeneous point-process entropy

Measures of entropy are primarily defined to address the randomness and regularity of a dynamical system given the analysis of time series originated by the observed system. Traditional algorithms provide a single value (or a set of values) within a predetermined time window. Therefore, given the experimental time series, these values represent averaged measures of the entire dynamics observed in that specific time window. However, a single estimation could not be sufficient to completely characterize system complexity in the face of non-stationary behavior. It is well-known that dynamical systems (particularly those associated with physiological processes) evolve and change at each moment in time. To overcome the limitations of the currently used entropy measures, within this study we use a recently introduced definition of approximate and sample entropy as instantaneous entropy measures of discrete system complexity.

The originality of the new definitions lies in the fact that they are fully embedded in the probabilistic framework of the inhomogeneous point-process theory, and introduce important differences to the mathematical formulation of the phase-space vectors and the definition of the distance between phase-space vectors.

Given the embedding dimension *m*, and time delay of the phase space *r(t)*, here we engage a novel tool for nonlinear dynamical systems by defining the distance *d[x(k),x(j)]* as the KS distance (Valenza et al., 2014d) (i.e., the maximum value of the absolute difference between two cumulative distribution functions) between the *IG_k_* and *IG_j_* probability distributions of μ_*RR*_(*t*_*k* + *k_n_*_) and μ_*RR*_(*t*_*j* + *k_n_*_) for *k_n_* = 0, 1, …, *m* − 1.

Then, it is possible to define (Valenza et al., 2014d):

$$C^{m}\left(r,t\right)={\left(N-m+1\right)}^{-1}\sum_{i=1}^{N-m+1}\ln\;C_{k}^{m}\left(r,t\right)$$

and obtain

$$ipApEn\left(m,r,N,t\right)=C^{m}\left(r,t\right)-C^{m+1}\left(r,t\right).$$

Note that the time-varying quantity *r(t)* is instantaneously expressed as *r*(*t*) 0.2σ_*RR*_(*t*) as previously suggested (Leistedt et al., 2011; Valenza et al., 2014d).

As the definition of the proposed entropy measure is fully embedded into the inhomogeneous point-process nonlinear framework, it is possible to obtain instantaneous tracking of the system complexity as *ipApEn (m, r, N, t)*. Of note, the definition of the *ipSampEn (m, r, N, t)* is slightly different (Valenza et al., 2014d). This second index does not take into account self-matches, and quantifies the time series regularity by slightly modifying the two equations above (Valenza et al., 2014d).

Both instantaneous assessments open the possibility of analyzing the proposed measures also in terms of variability of their evolution in time, which we refer to as *complexity variability framework* (Valenza et al., 2014d,f).

### Computation of other instantaneous linearly-derived parameters in the time and frequency domain

Aside from the calculation of the *ipApEn* and *ipSampEn* measures, the model described in the previous two paragraphs is also able to provide standard measures defined in the time and frequency domains gathered from the linear terms of Equation (2), as described in detail in Valenza et al. (2014e). In this way, it is possible to evaluate the effect of the sympathetic and parasympathetic nervous system activity interaction as a result of two main oscillatory components that are usually differentiated in the spectral profile (Acharya et al., 2006): (a) the high frequency (HF) band (0.15–0.40 Hz), which reflects the effects of respiration on HR, also referred to as respiratory sinus arrhythmia; (b) the low frequency (LF) band (0.04–0.15 Hz), which represents oscillations related to regulation of blood pressure and vasomotor tone including the 0.1 Hz fluctuation. In the time domain, the first and second order moments (μ_*RR*_ and σ^2^_*RR*_) of the IG distribution are calculated (Valenza et al., 2014d).

## Experimental results

**Figure 1:**
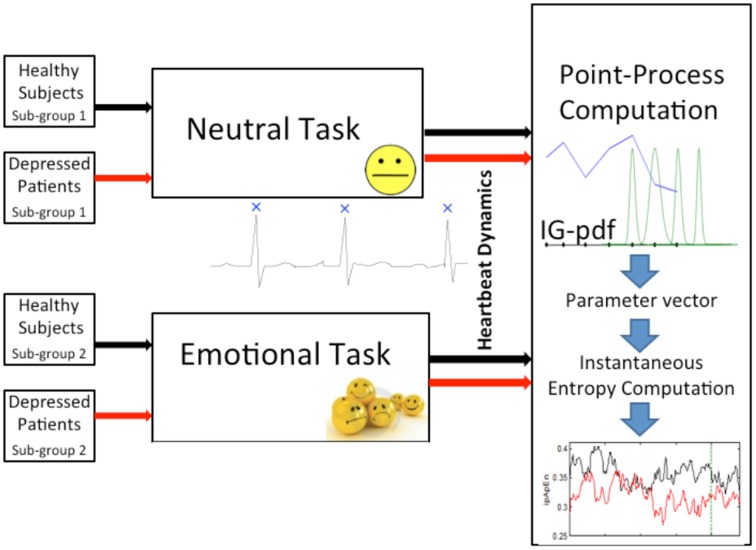
**Block scheme of the experimental protocol considered in this study**. Healthy subjects and depressed patients are split into two sub-groups, respectively. The first sub-group of healthy subjects and the first of depressed patients were randomly selected to undergo a neutral elicitation task, whereas the second respective sub-groups underwent an emotional elicitation task. Meanwhile, continuous ECG recording was performed in order to monitor the heartbeat dynamics of each subject/patient. Then, each RR interval series was modeled and processed through a point-process nonlinear model. Finally, starting from the time-varying parameter vector, the instantaneous entropy measures, ipApEn and ipSampEn, computation was performed.

**Figure 2:**
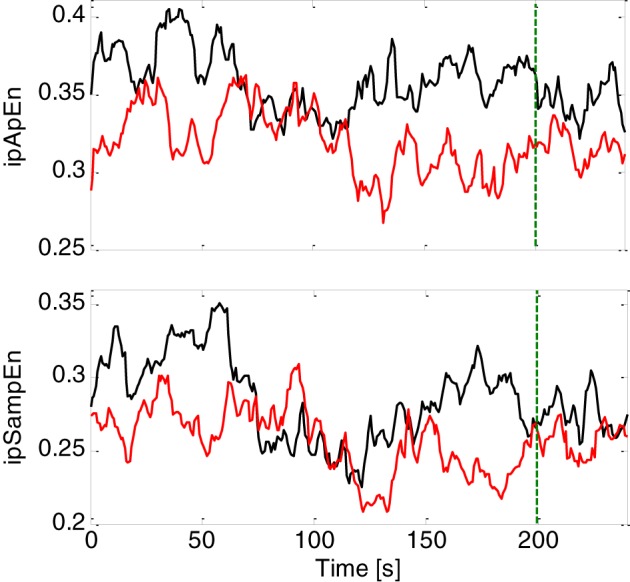
**Instantaneous complex heartbeat dynamics expressed as ipApEn (top panel) and ipSampEn (bottom panel) during the emotional elicitation task**. Values are averaged among healthy subjects (continuous black line) and patients with MD (continuous red line). The green dotter vertical line indicates the end of the audio-visual stimulus.

The primary goal of this study was to test the ability of instantaneous linear and complex nonlinear estimates of heartbeat dynamics in discriminating healthy subjects from the MD patients. We separated each task (neutral elicitation task or emotional elicitation task), and considered the median value over the whole acquisition for each subject (see Figure 1). The averaged instantaneous tracking of the complex heartbeat dynamics, expressed as *ipApEn* and *ipSampEn*, during the emotional elicitation task are shown in Figure 2. Values are averaged among healthy subjects and patients with MD.

The majority of the group samples resulted non-normally distributed (*p*-values gathered from Kolmogorov–Smirnov tests with null hypothesis of normality resulted <0.05, i.e., data are not normally distributed). Therefore, in order to average among the groups, each feature is expressed as its median value and its respective absolute deviation (i.e., for a feature X, X = median(X) ± MAD(X) where MAD(X) = |X − median(X)|). For each feature and for each experimental task, we tested the null hypothesis of having an equal median between the healthy and MD groups through the Mann–Whitney non-parametric test.

Our working hypothesis was that the degree of complexity of cardiovascular dynamics differs between normal and MD subjects. Since we used two measures to verify our hypothesis, we appropriately adjusted the statistical significance level to account for multiple comparisons in testing each individual measure. In the case of emotional elicitation, we observed that the difference is statistically significant for ipApEn while not for ipSampEn.

To confirm that this difference was due to nonlinear mechanisms rather than simply a reflection of linear changes on our nonlinear measures, we also applied the Mann–Whitney test on a batch of conventional HRV measures, such as μ_*RR*_, σ^2^_*RR*_, LF, HF, and LF/HF. None was statistically significant, even without accounting for multiple comparisons.

The statistics of each group and each instantaneous feature related to the neutral elicitation task are reported in Table 1, whereas those related to the emotional elicitation task are reported in Table 2.

**Table 1:** **Descriptive statistics of instantaneous heartbeat dynamics features during the neutral elicitation task**.

**Feature**	**Statistics**		
	**Healthy**	**Depressed**	***p*-value**
*ipSampEn*	0.286 ± 0.067	0.269 ± 0.023	0.970
*ipApEn*	0.345 ± 0.058	0.352 ± 0.046	0.447
μ_*RR*_	761.62 ± 83.42	798.21 ± 61.57	0.447
σ^2^_*RR*_	319.11 ± 191.27	521.90 ± 187.03	0.442
LF	485.53 ± 253.45	763.60 ± 408.17	0.056
HF	321.12 ± 225.92	228.04 ± 109.43	0.799
LF/HF	1.818 ± 1.186	1.768 ± 0.862	0.817

*p-values from the Mann–Whitney non-parametric test with null hypothesis of equal medians*.

**Table 2:** **Descriptive statistics of Instantaneous Heartbeat Dynamics features during the Emotional elicitation task**.

**Feature**	**Statistics**		
	**Healthy**	**Depressed**	***p*-value**
***ipApEn***	**0.361 ± 0.038**	**0.316 ± 0.041**	**0.016**
*ipSampEn*	0.298 ± 0.052	0.257 ± 0.041	0.212
μ_*RR*_	809.17 ± 102.25	792.40 ± 84.05	0.328
σ_2__*RR*_	596.71 ± 367.11	402.30 ± 209.61	0.093
LF	674.08 ± 417.99	616.29 ± 391.82	0.777
HF	518.34 ± 284.61	328.63 ± 214.38	0.054
LF/HF	1.068 ± 0.591	1.987 ± 0.751	0.095

*Bold indicates statistically significant indices*.

*p-values from the Mann–Whitney non-parametric test with null hypothesis of equal medians*.

**Figure 3:**
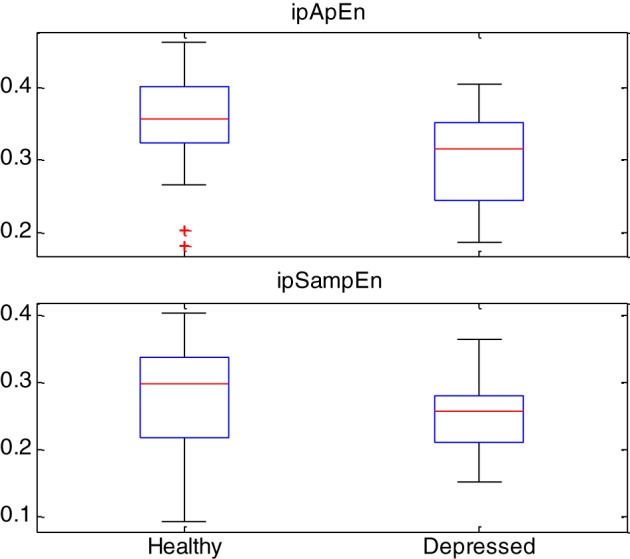
**Box-plot statistics of the instantaneous entropy measures ipApEn and ipSampEn gathered from the whole story with emotional task and grand-averaged among healthy subjects and depressed patients**. Red plus signs show outliers.

Box-plot statistics of the *ipApEn* and *ipSampEn* indices are shown in Figure 3.

Finally, we report that a *p* = 0.15 given by the Mann–Whitney non-parametric test was associated to the null hypothesis of having equal median age between the healthy and MD groups (age of healthy subjects: 21 ± 2; age of MD: 23 ± 2). This outcome confirms that our results are not affected by age differences between the groups.

## Discussions and conclusions

In this study, we aimed at proving the potential of computational methods based on nonlinear digital signal processing to provide critical information that could be used for devising automated diagnostic tools that can serve mental healthcare. In particular, we have presented the application of an advanced digital signal processing methodology in order to characterize a subject experiencing a mental disorder such as MD. Importantly, significant assessments can be performed considering only heartbeat dynamics elicited by time-varying emotional stimuli while estimating two recently proposed measures of complexity: *ipApEn* and *ipSampEn* (Valenza et al., 2014d).

The mathematical framework of these measures is based on point-process theory, which provides effective computational tools able to continuously estimate heartbeat dynamics without using any interpolation methods (Valenza et al., 2014e,f). This powerful, fully-parametric statistical method accounts for the probabilistic complex generative mechanism of the heartbeat by considering a quadratic Wiener–Volterra representation of the first order moment of a physiological plausible inverse-Gaussian statistics (Valenza et al., 2014d). Moreover, goodness-of-fit measures such as KS distance and autocorrelation plots quantitatively allow to verify the model fit and to choose the proper model order, thus addressing another open issue of current parametric approaches (Valenza et al., 2014d,e,f).

Our principal goal was to validate the utility of the presented computational framework by testing its ability in tracking nonlinear and non-stationary heartbeat dynamics of healthy subjects and MD patients undergoing affective elicitation. To this extent, we demonstrated that instantaneous complex heartbeat dynamics is indeed modulated by emotionally-relevant elicitations in patients with MD. These patients, in fact, exhibited lower *ipApEn* values than healthy subjects while elicited through arousing stimuli. These results are in agreement with the current literature showing that pathological mental states modulate cardiovascular complexity (Leistedt et al., 2011; Yang and Tsai, 2013; Valenza et al., 2014c). We further observed that this complexity modulation occurs only in case of emotionally-relevant stimuli. This is also in agreement with both clinical experience and the same research studies linking mood states, emotional regulation, and emotional response to ANS dynamics (Thayer et al., 1996; Carney et al., 2002; Iverson et al., 2005; Lanatà et al., 2012). For this reason, a possible approach to investigate mood recognition is to explore emotional changes provoked by external stimuli. From a modeling point of view, such approach interprets the cardiovascular system as coupled with the central nervous system and governed by nonlinear dynamical equations which can be characterized by means of a “perturbation” analysis, i.e., analysis before and after short-time emotional elicitation.

Our statistical analysis performed on the linear instantaneous features defined in the time and frequency domains suggests that, although sympatho-vagal dynamics can be affected by pathological mental states (Agelink et al., 2002; Acharya et al., 2006; Lemoult et al., 2012), the inter-subject variability is too high to allow such changes to be revealed through linear analysis. Conversely, the analysis on instantaneous complexity allowed to provide a much higher discriminating power. Our results further demonstrated that the differences in heartbeat complexity found among the healthy and MD groups are not biased by the age of the patients enrolled in the study nor by the r(t) values that are a function of the HRV standard deviation. Unlike other paradigms developed in the literature for characterizing human mental states, our approach is purely parametric, and the analytically-derived indices can be evaluated in a dynamic and instantaneous fashion. The presented point-process nonlinear analysis, in fact, represents a pioneering study in the field of mood assessment, as just being recently proposed for the characterization of heartbeat dynamics in bipolar patients (Wittchen and Jacobi, 2005). Future works will exploit further instantaneous nonlinear estimates such as high order statistics, and the instantaneous Lyapunov exponents (Valenza et al., 2014f).

From a physiological perspective, the inherent complexity of the cardiovascular system (e.g., the nonlinear neural signaling on the sinoatrial node) has been confirmed by our experimental results. Although the detailed physiology behind these complex dynamics has not been completely clarified, previous studies suggest that β-adrenoceptor system and cholinergic iper-driving might be involved (as seen in rats) (Beckers et al., 2006).

A further speculative interpretation of the results of this study points at the insular cortex and the pregenual anterior cingulate cortex (pgACC) as important affective areas of the brain. Recently presented neuroimaging data, in fact, revealed that these cortices are involved in various neuropsychiatric diseases such as mood disorders and, especially, MD, along with panic disorders, PTSD, obsessive-compulsive disorders, eating disorders, and schizophrenia (Nagai et al., 2007). Moreover, several glutamatergic mechanisms which alter the functional connectivity between pgACC and insular cortex are related to severe depression and depression severity (Horn et al., 2010). Since these particular brain regions are known to be responsible for crucial homeostatic (interoceptive) functions involving ANS signaling from the whole body (Craig, 2003), we speculate that such brain-heart signaling is significantly altered during mood disorders and can be revealed by time-varying complexity analysis of cardiovascular variability while emotionally eliciting the patients. This kind of elicitation activates the insular cortex, which has a crucial role in emotional processing (Nieuwenhuys, 2011), leading to altered heartbeat complex dynamics.

Because mood disorders produce an altered emotional response, the achievements reported in this study could have a relevant impact on mood disorder psychopathology diagnosis and treatment. Monitoring fast emotional responses as the result of fast stimulation times through instantaneous heartbeat dynamics could make a continuous evaluation of disorder progression possible, thus representing an important scientific advancement. On a final note, as emotional state is presently determined in a clinical setting using questionnaires with limited accuracy and quantitative power (Wittchen and Jacobi, 2005), a more automated and objective assessment using a noninvasive physiological and easy-to-monitor signal such as the ECG would provide improved inpatient and outpatient care, thereby significantly reducing the time and cost of mental healthcare.

### Conflict of interest statement

The authors declare that the research was conducted in the absence of any commercial or financial relationships that could be construed as a potential conflict of interest.
